# Early Effects of a Hypocaloric, Mediterranean Diet on Laboratory Parameters in Obese Individuals

**DOI:** 10.1155/2014/750860

**Published:** 2014-03-04

**Authors:** Marta Greco, Eusebio Chiefari, Tiziana Montalcini, Francesca Accattato, Francesco S. Costanzo, Arturo Pujia, Daniela Foti, Antonio Brunetti, Elio Gulletta

**Affiliations:** ^1^Department of Health Sciences, Magna Græcia University of Catanzaro, Viale Europa (Località Germaneto), 88100 Catanzaro, Italy; ^2^Department of Medical and Surgical Sciences, Magna Græcia University of Catanzaro, Viale Europa (Località Germaneto), 88100 Catanzaro, Italy; ^3^Department of Clinical and Experimental Medicine, Magna Græcia University of Catanzaro, Viale Europa (Località Germaneto), 88100 Catanzaro, Italy

## Abstract

Calorie restriction is a common strategy for weight loss in obese individuals. However, little is known about the impact of moderate hypocaloric diets on obesity-related laboratory parameters in a short-term period. Aim of this study was to evaluate the variation of laboratory biomarkers in obese individuals following a Mediterranean, hypocaloric (1400–1600 Kcal/die) diet. 23 obese, pharmacologically untreated patients were enrolled and subjected to the determination of anthropometric variables and blood collection at baseline, 1 and 4 months after diet initiation. After 4 months of calorie restriction, we observed a significant decrease in body weight and BMI (both *P* < 0.0001), insulin (*P* = 0.037), HOMA-IR (*P* = 0.026), leptin (*P* = 0.008), and LDH (*P* = 0.023) and an increase in EGF (*P* = 0.013). All these parameters, except LDH, varied significantly already at 1 month after diet initiation. Also, lower levels of insulin (*P* = 0.025), leptin (*P* = 0.023), and EGF (*P* = 0.035) were associated with a greater (>5%) weight loss. Collectively, our data support a precocious improvement of insulin and leptin sensitivity after a modest calorie restriction and weight reduction. Moreover, EGF and LDH may represent novel markers of obesity, which deserve further investigations.

## 1. Introduction

Obesity, a chronic disorder whose prevalence is increasing in adults, adolescents, and children, is now considered a worldwide epidemic and an important issue in the health care [1, 2]. In many populations, the prevalence of overweight and obesity has rapidly increased in the past 20 years. In the USA, more than 35% of adults and almost 17% of young people were classified as obese in 2009-2010 [3], and the lifetime risk of developing overweight or obesity was estimated to be roughly 50 and 25%, respectively [4].

Obesity predisposes to a number of pathological conditions, including cardiovascular diseases [5, 6], type 2 diabetes [7, 8], metabolic syndrome [9], nonalcoholic fatty liver disease [10], certain types of cancer [11, 12], obstructive sleep apnea [13], osteoarthritis [14], and asthma [15], and increases the risk of premature death [16], leading to elevated health care costs worldwide [17].

Factors predisposing to obesity include excessive food intake, lack of physical activity, and genetic susceptibility [18]. However, obesity is a heterogeneous condition, and intra-abdominal accumulation of fat tissue has been shown to have a higher impact on the development of cardiometabolic risk factors and related disorders than total excessive adiposity [19]. Increased free fatty acid availability, the release of proinflammatory cytokines and adipokines from adipose tissue, hepatic insulin resistance and inflammation, and the consequent dyslipidemia are among the many metabolic disorders associated with this condition. Hormonal status and drug treatments may also play an important role [20]. Also, increased levels of some hemostatic factors have been described that may explain the prothrombotic risk of obese patients [21].

Although surgical [22] and pharmacological therapies [23] have been shown to be useful, hypocaloric diet still represents the main and essential step in the treatment of obesity, together with increased physical activity and changes in lifestyle [24]. Weight loss, in fact, is an important goal and has proved to be beneficial in preventing health risks related to obesity [25, 26]. In this context, many hypocaloric diets are available that can be potentially recommended, including Mediterranean diet [27].

The association between the effects of a low-calorie diet and obesity is however very complex and not yet fully understood. In particular, little is known about the pathogenetic mechanisms and their putative markers that link potential benefits of weight loss, in a short-term period, to the asymptomatic, obese patients.

To elucidate possible relationships between obesity, diet, and weight loss, herein, we evaluated the variation, at short time intervals, of some anthropometric and laboratory parameters in obese individuals, subjected to a Mediterranean, low-calorie diet. In the effort to identify useful precocious markers in the follow-up of these patients, we analysed laboratory parameters correlated with the pathophysiological changes linked to obesity, including indexes of insulin resistance, proinflammatory and prothrombotic markers, adipocytokines, and lipid profile.

## 2. Materials and Methods

### 2.1. Patients and Study Design

We designed a prospective, descriptive study in the routine management of patients with obesity.

52 patients that attended the Clinical Nutrition Operative Unit of our University and were not taking any medications were initially enrolled from January 2012 to May 2013. Due to the high drop-off rate, 23 patients (17 females and 6 males), age 52 (46–61 yrs) and average body mass index (BMI) 37 kg/m^2^, completed the study. Obesity was classified, according to the WHO guidelines, in grade I (BMI: 30–34.9 kg/m^2^), grade II (BMI: 35–39.9 kg/m^2^), and severe obesity (BMI: ≥40 kg/m^2^). Percentage of fat mass and percentage of lean mass were established at the enrollment by bioelectrical impedance analysis using the application Bodygram Pro (Akern, Pontassieve, FI, Italy). Any pharmacological treatment or intercurrent diseases during follow-up were assumed as exclusion criteria. Informed consent was obtained by each patient enrolled in the study.

After careful consideration of global nutrition, a personalized, Mediterranean hypocaloric diet (1400–1600 kcal/die) was prescribed, aiming at the achievement of a moderate weight loss. The proposed food plan included carbohydrates (55% of total calories), proteins (20% of total calories), and mostly mono- and polyunsaturated fats (25% of total calories). The intake of cholesterol in the diet accounted for less than 300 mg/day, while fibers were equal to 25–30 g/day. The diet was also balanced in terms of micronutrients intake.

Patients underwent two sessions of behavioral dietary counseling: one at the first visit, which focused on notions of nutrition, dietary errors, suggestions on food preparation techniques, and the importance of associating physical exercise (30 minutes of speed walking, 3 days/week) with diet therapy, and a second session at 1 month from dietary treatment, to reinforce adherence to diet. During the first visit, patients were also subjected to anthropometric determinations (body weight and height). During the first visit, patients were also subjected to anthropometric determinations, including body weight and height for BMI calculation, and blood collection. Fasting blood samples were collected from all participants with no caloric intake for at least 12 h, and the following biochemical analyses were performed: glucose, insulin, cystatin C, adipokines (leptin, adiponectin, resistin, and visfatin), prothrombin time (PT), activated partial thromboplastin time (aPTT), fibrinogen, plasminogen activator inhibitor-1 (PAI-1), triglycerides, total cholesterol, and HDL cholesterol, *γ*-glutamyl-transferase (*γ*GT), aspartate aminotransferase (AST), alanine aminotransferase (ALT), lactate dehydrogenase (LDH), high-sensitivity C reactive protein (hsCRP), tumor necrosis factor *α* (TNF*α*), interleukins (IL): IL-1*α*, IL-1*β*, IL-2, IL-4, IL-6, IL-8, and IL-10, monocyte chemoattractant protein-1 (MCP-1), vascular endothelial growth factor (VEGF), and epidermal growth factor (EGF). Insulin resistance was calculated with the homeostatic model assessment method of insulin resistance (HOMA-IR) [28]. The same patients were visited and subjected to the same blood determinations after 1 and 4 months from dietary treatment. We established a cut-off of 5% at 4 months to indicate the efficacy of treatment.

### 2.2. Blood Sample Collection and Routine Work-Up

Blood was collected, after 12–14 h fasting, by antecubital venous puncture. Serum or plasma samples were obtained by centrifugation. Routine analyses, including blood count, PT, PTT and fibrinogen, fasting glucose, total cholesterol, HDL and LDL, triglycerides, AST, ALT, *γ*GT, and LDH, were obtained from fresh samples, whereas aliquots of serum or citrated plasma were frozen at −80° for subsequent laboratory determinations (insulin, adipokines, cytokines, hsCRP, cystatin C, and PAI-1).

Blood count analysis was performed using ADVIA 2120 (Siemens, USA); PT, aPTT, and fibrinogen were performed on BCS XP (Siemens, USA); blood glucose, total cholesterol, HDL and LDL, triglycerides, AST, ALT, *γ*GT, and LDH were performed on Cobas 6000 (Roche, Switzerland). All the above-mentioned assays were carried out according to the manufacturer's instructions.

Reference values of each measured analyte are reported in the supplementary table (see Supplementary Material available online at http://dx.doi.org/10.1155/2014/750860).

### 2.3. Measurements of Serum Cystatin C and hsCRP

The determinations of serum concentrations of cystatin C and hsCRP were carried out using a nephelometer BNII (Siemens, USA) by immunonephelometric methods, according to the manufacturer's instructions (Penia N Latex Cystatin C kit and CardioPhase hsCRP kit, Siemens, USA, resp.).

### 2.4. Measurement of Serum Cytokines

The serum concentrations of 12 different cytokines and growth factors (IL-1*α*, IL-1*β*, IL-2, IL-4, IL-6, IL-8, IL-10, IFN*γ*, TNF*α*, MCP-1, VEGF, and EGF) were simultaneously determined using the biochip analyser Evidence Investigator (Randox Labs, UK) and the “Cytokine Array I and High sensitivity” kit, Randox, UK, according to the manufacturer's instructions. Briefly, the principle of this multianalyte testing relies on a sandwich ELISA, in which the analytes of interest are captured by specific antibodies bound to discrete regions of the surface chemistry of the biochip; horseradish peroxidase (HRP) labeled secondary antibodies, which specifically recognize the analytes, trigger a luminol-based electrochemiluminescent signal emission, registered by a CCD camera and quantified by a software.

### 2.5. Measurement of Serum Insulin and Adipokines

The serum concentration of insulin was measured by the ADVIA Centaur Immunoassay system (Siemens, USA), using a chemiluminescent immunoassay. Serum adipokines were determined by sandwich ELISA assays on the automated ELISA analyser Triturus (Grifols, USA), with the kits produced by Mediagnost, Germany (Adiponectin E09, sensitive leptin E077, resistin E50). Visfatin was determined by Visfatin C-Terminal Human, EIA Kit (Phoenix).

### 2.6. Statistical Analysis

Each quantitative trait was tested for normality using the Shapiro-Wilk normality test and, when required, it has been log-transformed. Any continuous variable was indicated as median and interquartile range. The nonparametric Friedman and Wilcoxon tests were used to evaluate the differences between the intragroup and anthropometric variables and laboratory parameters during the time of the study. A significance level of *P* < 0.05 was set for a type I error in all analyses. Multivariate analysis was performed by incorporating age and sex as covariates using the linear regression procedure. All statistical procedures were performed by using the SPSS 20.0 software for Mac.

## 3. Results

The characteristics of the population studied are summarized in Table 1. Most participants (74%) were women, and the mean age was 52 years. At baseline, the population enrolled was affected by grade I, grade II, and severe obesity (34.8%, 43.5%, 21.7%, resp.), and the mean BMI was 37 kg/m^2^. Data on body composition indicate the contribution of adiposity to body weight. Comparison of all the parameters examined at time of 0, 1, and 4 months from diet treatment is shown in Table 2. During the follow-up, body weight and BMI significantly decreased (*P* < 0.001) after calorie restriction, with an average reduction of body weight of 3.4% at 1 month and 5.8% at 4 months after dieting. After the 4-month dietary intervention, in fact, body weight loss redistributed the status of patients and their proportions, as overweight (17.4%) and grade I (34.8%) and grade II (26.1%) obese, while the percentage of patients with severe obesity had no variation (21.7%).

Weight loss led to an improvement in insulin sensitivity, as indicated by a decrement of both insulin (*P* = 0.037) and HOMA-IR index (*P* = 0.026). LDH showed a decrement after 4 months of treatment (*P* = 0.023), whereas leptin was the biomarker that varied more significantly throughout the whole time period (*P* = 0.008). Also, to note, EGF showed a significant increase (*P* = 0.013). Some other analytes showed an interesting trend of variability. In particular, a decremental trend has been observed for IL-6 and VEGF and for prothrombotic risk parameters, such as fibrinogen and PAI-1 (Table 2). To strengthen the precocious variation of the parameters examined, we compared all the variables after only one month of dietary intervention. Statistical analysis showed significant variations of insulin, HOMA-IR, leptin, resistin, EGF, and total cholesterol, confirming leptin as the most sensitive marker (Table 3).

Then, we analyzed the effect of weight loss on biochemical parameters after 4 months, compared subjects who obtained a decrease in their body weight greater than 5% with those who did not, and arbitrarily used a weight loss >5% as a group variable. Notably, we observed a more significant variation of insulin (*P* = 0.025), leptin (*P* = 0.023), and EGF (*P* = 0.035) in patients achieving a greater body weight loss (Table 4).

## 4. Discussion

Diet represents the most widespread tool to reduce weight in the obese individuals [29]. It has been shown that even a moderate weight loss of about 10% or less contributed to several health advantages that include improvement in metabolic parameters, reduction of blood pressure, and increase in longevity [25, 26]. Also, there are circumstantial lines of evidence that calorie restriction improves insulin sensitivity and reduces systemic inflammation [30–33].

Several reports [27, 34–36] have emphasized the benefits of weight loss in obese individuals by evaluating physical and biochemical markers after at least 6 months from diet introduction, whereas little is known about possible earlier changes of these indexes. In particular, by comparing different hypocaloric diets, the Mediterranean diet has proved to be associated with a greater improvement of insulin sensitivity parameters [27]. The innovative aspects of our study consisted in the short-term assessment, in obese subjects, of clinical and laboratory parameters after a hypocaloric, Mediterranean diet and the achievement of a generally modest weight loss.

In our study, the efficacy of dietary treatment was proved by an average reduction of body weight of 3.4% at 1 month and 5.8% at 4 months and by a statistically significant reduction in BMI. Most studies conducted so far have considered a minimum of 5% body weight loss indispensable to obtain beneficial effects [25, 26, 37]. Interestingly, our findings show that, even after a smaller decrement of body weight, diet is associated with a precocious, statistically significant reduction of insulin levels and HOMA-IR index that are compatible with an early improvement of insulin sensitivity. We cannot however exclude or quantify the possible contribution of physical activity to this issue.

We also examined cytokines and adipokines for their link to the pathophysiology of obesity and insulin resistance. It is well known that the adipose tissue is an endocrine organ with a wide, biologically active secretome [38, 39] and that the adipose tissue of obese individuals is characterized by an increased expression and/or secretion of several proinflammatory cytokines, as well as of adipokines known to promote inflammation, atherogenesis, and insulin resistance, whereas the biosynthesis of anti-inflammatory, antiatherogenic, and insulin-sensitising adipokines, such as adiponectin, is decreased [40–42].

Several studies have shown that increased circulating leptin levels and increased adiposity are associated with leptin resistance and that this condition can contribute to the onset and/or maintenance of obesity [43–46]. In our study, we observed, throughout the follow-up, a significant decrease in circulating levels of leptin, which showed a statistically significant positive correlation with both BMI and insulin. Although insulin and leptin resistance have been widely described in the obese subjects, the relationships between these two hormones are complex and not fully understood, and evidence exists that they are associated with body fat through different mechanisms [47, 48]. No statistical variations of other adipokines or cytokines have been observed after dietary treatment in the whole time period of the study. Other studies with comparable or even greater sample size had obtained similar results and emphasized a statistically significant decrement in leptin levels following diet [49–51]. Leptin, but not adiponectin, resistin, RBP-4, or cytokines, has been correlated with calorie restriction and weight loss in a recent study [52], while an improvement in adipokine profile was observed in severely obese women, only after a 5 to 10% weight loss [37].

Based on previous findings and on the consideration that BMI is a good predictor of cardiovascular disease risk [53, 54], we hypothesized that weight loss could be associated with an improvement in hemostatic parameters. Due to the small sample size, our results showed nonstatistically significant differences, as also reported by other works that investigated an even greater number of patients [55, 56].

An intriguing and apparently equivocal finding in our study is the precocious increase of circulating EGF levels after diet introduction, while EGF decreases in the patient group that had lost >5% of body weight, in association with an improvement in insulin sensitivity. Salivary glands are the major source of circulating EGF, a growth factor implicated in the control of cell proliferation and differentiation [57]. During fasting, salivary and plasma EGF physiologically increase [58] to inhibit gastric acid secretion and to preserve the oroesophageal and gastrointestinal mucosal integrity. We hypothesize that the increase of EGF observed in our study is coherent with the food restriction state and with the intestinal adaptation to diet. The increase in EGF may exert insulin-like biological activities in tissues expressing high levels of EGF receptors (EGFR), such as fat and skeletal muscle [59]. By binding to EGFR, a receptor belonging to the tyrosine kinase receptor family, like the insulin receptor [60], EGF can amplify the downstream signaling of insulin through the recruitment of additional PI3-kinase pools in tissues, in which both cognate receptors and the downstream signaling molecules are all abundantly expressed, leading to an EGF-induced translocation of GLUT4 on the plasma membranes and the stimulation of glucose uptake in target tissues [59]. These molecular mechanisms are particularly important in insulin-resistant states, including obesity, in which the increase of EGF due to food restriction may trigger insulin-like compensatory mechanisms. It is also known that insulin regulates EGF expression [61]. This explains the parallel decrease of both insulin and EGF in patients who achieved a more consistent weight loss, in which insulin sensitivity has been improved or reestablished.

LDH is an intracellular enzyme that is released into the bloodstream when tissues are destroyed or injured. Therefore, LDH is considered an important clinical marker of tissue or cell integrity. In the obese, the adipose tissue is dysfunctional [62, 63] and exhibits a reduced capacity to store and retain nonesterified fatty acids (NEFA), leading to an increase in NEFA circulating levels, thereby promoting the development of lipotoxicity in peripheral tissues [64]. In this study, we observed a significant reduction of circulating levels of LDH following diet, a condition compatible with an attenuation of the dysfunctional, proinflammatory mechanisms that characterizes obesity.

## 5. Conclusions

On the whole, our study confirms the efficacy of a moderate hypocaloric Mediterranean diet to improve insulin sensitivity in obese patients. The novelty of our findings is that calorie restriction determines a precocious variation of both insulin and leptin even after achieving a modest weight loss. In addition to these traditional laboratory parameters, LDH and EGF have emerged as novel obesity-related markers, pathophysiologically linked to obesity and insulin resistance, that deserve further attention. In particular, the precocious increment of EGF might be clinically useful as an early predictor for a positive evolution of the metabolic response following calorie restriction.

## Supplementary Material

Reference values of biochemical traits.

## Figures and Tables

**Table 1:** Demographic and anthropometric features of obese patients at the enrollment.

	Patients
Race	Caucasian
*N*	23
Female	17
Age (yrs)	52 (46–61)
Height (cm)	155 (153–167)
BMI (Kg/m^2^)	37 (32–39)
FM (%)	43.5 (34.5–46.0)
LM (%)	37.0 (33.0–43.7)
FM/LM	1.2 (0.8–1.4)

FM: fat mass; LM: lean mass, as calculated by bioelectrical impedance analysis. Continuous variables are expressed as median and quartiles.

**Table 2:** Comparison of demographic, anthropometric, clinical, and biochemical features of obese patients at baseline and during follow-up.

	Baseline	1 month	4 months	*P*
Weight (Kg)	91 (82–112)	87 (83–101)	87 (78–98)	<0.001
BMI (Kg/m^2^)	37 (32–39)	36 (31–38)	35 (31–38)	<0.001
Total cholesterol (mg/dL)	199 (172–225)	190 (151–222)	209 (168–218)	0.249
HDL (mg/dL)	50 (38–57)	44 (39–53)	49 (41–57)	0.073
LDL (mg/dL)	131 (95.0–160.0)	117 (95.0–153.0)	120 (95.0–149)	0.444
Triglycerides (mg/dL)	105 (66–169)	111 (76–132)	95 (83–149)	0.420
Fasting glucose (mg/dL)	97 (91–107)	96 (91–99)	94 (87–104)	0.168
Basal insulin (*µ*U/mL)	18.0 (13.0–27.0)	15.0 (10.0–20.0)	14.0 (10.0–28.0)	0.037
HOMA-IR	4.0 (3.0–6.0)	4.0 (2.0–5.0)	3.0 (3.0–6.0)	0.026
*γ*GT (U/L)	22.0 (15.0–44.0)	21.0 (14.0–40.0)	19.0 (13.0–65.0)	0.299
AST (U/L)	23.0 (17.0–27.0)	21.0 (18.0–24.0)	19.0 (16.0–24.0)	0.238
ALT (U/L)	27.0 (19.0–34.0)	25.0 (18.0–35.0)	22.0 (17.0–33.0)	0.220
LDH (U/L)	345 (326–397)	350.0 (329–403)	323 (296–376)	0.023
Adiponectin (*µ*g/mL)	5.0 (3.0–7.0)	4.0 (3.0–6.0)	5.0 (3.0–7.0)	0.923
Leptin (ng/mL)	47.0 (31.0–70.0)	35.0 (22.0–46.0)	36.0 (27.0–44.0)	0.008
Resistin (ng/mL)	5.0 (4.0–6.0)	5.0 (5.0–7.0)	5.0 (4.0–7.0)	0.267
Visfatin (ng/mL)	7.0 (5.0–8.0)	7.0 (6.0–9.0)	7.0 (6.0–9.0)	0.559
Cystatin C (mg/dL)	0.79 (0.71–0.85)	0.78 (0.67–0.85)	0.78 (0.72–0.85)	0.640
PT (%)	102 (98–104)	103 (96–104)	104 (99–110)	0.211
aPTT (sec)	27 (26–28)	28 (27–30)	27 (25–29)	0.001
Fibrinogen (mg/dL)	325 (274–369)	324 (287–349)	308 (260–341)	0.676
PAI-1 (U/mL)	5.0 (2.0–6.0)	3.0 (1.0–6.0)	3.0 (1.0–4.0)	0.610
hsCRP (mg/dL)	2.8 (1.8–7.0)	2.8 (1.61–5.2)	4.0 (1.2–6.4)	0.568
IL-2 (pg/mL)	0.0 (0.0–5.0)	3.0 (0.0–5.0)	3.0 (0.0–6.0)	0.154
IL-4 (pg/mL)	0.0 (0.0–2.0)	0.0 (0.0–2.0)	0.0 (0.0–0.0)	0.409
IL-6 (pg/mL)	1.3 (1.0–2.0)	1.3 (1.0–2.0)	1.0 (1.0–2.0)	0.394
IL-8 (pg/mL)	10.0 (7.0–28.0)	14.0 (6.0–38.0)	14.0 (10.0–25.0)	0.535
IL-10 (pg/mL)	0.0	0.0	0.0	—
VEGF (pg/mL)	193 (98–361)	178 (101–283)	174 (90–267)	0.568
IFN*γ* (pg/mL)	2.0 (0.0–4.0)	2.0 (0.0–4.0)	3.0 (0.0–6.0)	0.486
TNF*α* (pg/mL)	3.0 (3.0–5.0)	3.0 (3.0–6.0)	4.0 (3.0–7.0)	0.867
IL-1*α* (pg/mL)	0.0	0.0	0.0	—
IL-1*β* (pg/mL)	0.8 ± 2.0*	0.7 ± 2.3*	0.8 ± 2.7*	0.662
MCP-1 (pg/mL)	351 (206–395)	352 (232–515)	367 (214–485)	0.302
EGF (pg/mL)	86 (68–130)	114 (84–144)	97 (68–139)	0.013

Nonparametric Friedman test has been used for comparisons at three times. All variables are expressed as median and quartiles. *These data are expressed as average ± SD.

**Table 3:** Significant variations of anthropometric, clinical, and biochemical features of obese patients after one month of calorie restriction.

	Baseline	1 month	*P*
Weight (Kg)	91 (82–112)	87 (83–101)	<0.001
BMI (Kg/m^2^)	37 (32–39)	36 (31–38)	<0.001
Basal insulin (µU/mL)	18.0 (13.0–27.0)	15.0 (10.0–20.0)	0.011
HOMA-IR	4.0 (3.0–6.0)	4.0 (2.0–5.0)	0.006
Leptin (ng/mL)	46.0 (31.0–70.0)	35.0 (22.0–46.0)	<0.001
Resistin (ng/mL)	5.0 (4.0–6.0)	5.0 (5.0–7.0)	0.017
Total cholesterol (mg/dL)	199 (172–225)	190 (148–220)	0.047
EGF (pg/mL)	86 (68–130)	114 (84–144)	0.018

Nonparametric Wilcoxon test has been used for comparisons at two times. All variables are expressed as median and quartiles.

**Table 4:** Effect of weight loss on biochemical parameters.

	Weight loss >5%	Weight loss ≤5%	Beta	*t*	*P*
Total cholesterol (mg/dL)	206 (184–226)	177 (146–218)	0.338	1.699	0.106
HDL (mg/dL)	52 (44–68)	49 (45–54)	0.075	0.321	0.752
Triglycerides (mg/dL)	89 (79–154)	101 (71–134)	0.020	0.081	0.936
Fasting glucose (mg/dL)	95 (88–119)	94 (86–103)	0.300	1.181	0.252
Basal insulin (*µ*U/mL)	10.0 (8.7–15.7)	24.0 (12.0–28.5)	−0.506	−2.430	0.025
HOMA-IR	3.0 (2.0–3.2)	5.0 (3.0–6.0)	−0.435	−2.040	0.056
*γ*GT (U/L)	19.5 (14.2–56.0)	17.0 (11.0–81.0)	0.123	0.487	0.632
AST (U/L)	20.5 (15.7–24.2)	18.0 (16.0–24.0)	0.144	0.646	0.526
ALT (U/L)	19.0 (17.2–32.0)	23.0 (14.5–33.5)	0.329	1.317	0.203
LDH (U/L)	323 (298–389)	334 (295–363)	−0.141	−0.587	0.564
Adiponectin (*μ*g/mL)	6.0 (4.0–7.25)	4.0 (3.0–5.0)	0.298	1.164	0.259
Leptin (ng/mL)	31.5 (19–40.0)	41.0 (31.5–53.5)	−0.575	−2.478	0.023
Resistin (ng/mL)	5.0 (4.0–8.0)	6.0 (4.0–9.0)	−0.258	−0.940	0.359
Visfatin (ng/mL)	7.5 (6.0–10.0)	7.0 (6.0–8.5)	0.088	0.353	0.729
Cystatin C (mg/dL)	1.0 (1.0-1.0)	1.0 (1.0-1.0)	—	—	—
PT (%)	105 (102–111)	103 (97–108)	0.079	0.321	0.752
aPTT (sec)	27 (25–28)	27 (25–29)	−0.121	−0.554	0.586
Fibrinogen (mg/dL)	297 (261–346)	319 (244–339)	−0.068	−0.271	0.790
PAI-1 (U/mL)	2.5 (1.0–4.5)	3.0 (1.5–4.0)	0.010	0.038	0.970
hsCRP (mg/dL)	4.5 (1.0–9.2)	4.0 (2.0–5.5)	0.014	0.065	0.949
IL-2 (pg/mL)	3.5 (0.0–5.2)	3.0 (0.0–6.0)	0.001	0.002	0.999
IL-4 (pg/mL)	0.0 (0.0–2.0)	0.0 (0.0–2.0)	—	—	—
IL-6 (pg/mL)	1.0 (1.0–2.0)	1.0 (1.0–2.0)	0.058	0.256	0.801
IL-8 (pg/mL)	15.0 (12.5–28.2)	12.0 (9.0–23.0)	0.070	0.298	0.769
IL-10 (pg/mL)	0.0	0.0	—	—	—
VEGF (pg/mL)	230 (69–347)	157 (100–238)	−0.025	−0.105	0.917
IFN*γ* (pg/mL)	4.0 (0.7–9.7)	2.0 (0.0–4.0)	0.318	1.159	0.271
TNF*α* (pg/mL)	4.0 (2.7–7.2)	5.0 (4.0–7.5)	−0.191	−0.887	0.386
IL-1*α* (pg/mL)	0.0	0.0	—	—	—
IL-1*β* (pg/mL)	1.4 ± 4.2*	0.3 ± 0.7*	—	—	—
MCP-1 (pg/mL)	372 (228–547)	338 (203–444)	0.243	1.111	0.280
EGF (pg/mL)	77 (54.5–106.2)	121 (87–156)	−0.468	−2.273	0.035

Multivariate linear regression analysis has been adjusted for gender and age. All variables are expressed as median and quartiles.*These data are expressed as average ± SD.
