# Supplementary material for: Early Effects of a Hypocaloric, Mediterranean Diet on Laboratory Parameters in Obese Individuals
Source: Mediators Inflamm. 2014 Mar 4;2014:750860. doi: 10.1155/2014/750860 (PMC3960747; doi:10.1155/2014/750860)
Supplement: Supplementary file 1 — Reference values of biochemical traits. [file 750860.f1.pdf]

Supplementary Table. Reference values of biochemical traits.

	Reference values
Total cholesterol (mg/dL)	<200
HDL (mg/dL)	>35
Triglycerides (mg/dL)	<150
Fasting Glucose (mg/dL)	70-110
Basal insulin (μU/mL)	4-25
HOMA-IR	<2.5
γGT (U/L)	8-61
AST (U/L)	<38
ALT (U/L)	<41
LDH (U/L)	240-480
Adiponectin (μg/mL)	>10
Leptin (ng/mL)	<40
Resistin (ng/mL)	>4
Visfatin (ng/mL)	>2
Cystatin C (mg/dL)	0.53-0.95
PT (%)	70-120
aPTT (sec)	24-35
Fibrinogen (mg/dL)	200-400
PAI-1 (U/mL)	0.3-3.5
hsCRP (mg/dL)	1.69-2.87
IL-2 (pg/mL)	0.0-15.2
IL-4 (pg/mL)	0.0-20.3
IL-6 (pg/mL)	0.0-5.6
IL-8 (pg/mL)	1.9-17.4
IL-10 (pg/mL)	0.0-6.3
VEGF (pg/mL)	15.5-431.3
IFNγ (pg/mL)	0.0-13.6
TNFα (pg/mL)	0.0-13.3
IL-1α (pg/mL)	0.0-2.8
IL-1β (pg/mL)	0.0-2.4
MCP-1 (pg/mL)	70.5-209.3
EGF (pg/mL)	6.7-170.2
